# Enhancement of the *in vivo* persistence and antitumor efficacy of CD19 chimeric antigen receptor T cells through the delivery of modified TERT mRNA

**DOI:** 10.1038/celldisc.2015.40

**Published:** 2015-12-08

**Authors:** Yun Bai, Shifeng Kan, Shixin Zhou, Yuting Wang, Jun Xu, John P Cooke, Jinhua Wen, Hongkui Deng

**Affiliations:** 1 Department of Cell Biology and Stem Cell Research Center, School of Basic Medical Sciences, Center for Molecular and Translational Medicine, Peking University Health Science Center, Beijing, China; 2 The MOE Key Laboratory of Cell Proliferation and Differentiation, College of Life Sciences, Peking-Tsinghua Center for Life Sciences, Peking University, Beijing, China; 3 Shenzhen Stem Cell Engineering Laboratory, Key Laboratory of Chemical Genomics, Peking University Shenzhen Graduate School, Shenzhen, China; 4 Department of Cardiovascular Sciences, Center for Cardiovascular Regeneration, The Methodist Hospital Research Institute, Houston, TX, USA

**Keywords:** hTERT, CD19 CAR T cells, modified mRNA

## Abstract

Chimeric antigen receptor T cell immunotherapy is a promising therapeutic strategy for treating tumors, demonstrating its efficiency in eliminating several hematological malignancies in recent years. However, a major obstacle associated with current chimeric antigen receptor T cell immunotherapy is that the limited replicative lifespan of chimeric antigen receptor T cells prohibits the long-term persistence and expansion of these cells *in vivo*, potentially hindering the long-term therapeutic effects of chimeric antigen receptor T cell immunotherapy. Here we showed that the transient delivery of modified mRNA encoding telomerase reverse transcriptase to human chimeric antigen receptor T cells targeting the CD19 antigen (CD19 chimeric antigen receptor T cells) would transiently elevate the telomerase activity in these cells, leading to increased proliferation and delayed replicative senescence without risk of insertion mutagenesis or immortalization. Importantly, compared to conventional CD19 chimeric antigen receptor T cells, after the transient delivery of telomerase reverse transcriptase mRNA, these CD19 chimeric antigen receptor T cells showed improved persistence and proliferation in mouse xenograft tumor models of human B-cell malignancies. Furthermore, the transfer of CD19 chimeric antigen receptor T cells after the transient delivery of telomerase reverse transcriptase mRNA enhanced long-term antitumor effects in mouse xenograft tumor models compared with conventional CD19 chimeric antigen receptor T cell transfer. The results of the present study provide an effective and safe method to improve the therapeutic potential of chimeric antigen receptor T cells, which might be beneficial for treating other types of cancer, particularly solid tumors.

## Introduction

Chimeric antigen receptor (CAR) T-cell immunotherapy has recently emerged as a promising approach to treat tumors [1, 2], and this therapeutic strategy has shown significant advantages over traditional T-cell immunotherapy. The expression of CAR in T lymphocytes confers the ability to recognize specific tumor antigens [3]. Furthermore, CARs redirect T-cell specificity in an HLA-independent manner, thereby eliminating the need to consider HLA restriction and overcoming some tumor escape mechanisms [4]. Importantly, several recent clinical trials have shown that CAR T cells targeted to the CD19 antigen efficiently induce complete remission in patients with acute or chronic lymphoblastic leukemias [5–7], suggesting the potential of CAR T-cell immunotherapy in eliminating cancer cells.

One major problem of current CAR T-cell immunotherapy is that T lymphocytes have limited replicative lifespans [8, 9], which potentially influences the long-term antitumor effect of CAR T-cell immunotherapy. Similar to most somatic human cells, T lymphocytes have a limited replicative lifespan, referred to as replicative senescence [10, 11]. Previous studies have shown that the therapeutic efficacy of adoptive T-cell transfer is correlated with the ability of T cells to proliferate and survive *in vivo* [12, 13]. It has been reported that T cells with minimal differentiation (non-senescent) and non-exhaustion have the highest antitumor activity because replicative senescence in T cells results in the loss of proliferative capacity and functional impairment with subsequent physical deletions [14, 15]. Therefore, the replicative senescence of T cells presents a major barrier for the clinical application of CAR T-cell immunotherapy, requiring novel strategies to solve this problem.

Among various factors involved in the regulation of the lifespan of T cells, telomeres are a major factor directly associated with the senescence of T cells [16, 17]. In most human cell types, including T cells, telomeres lose a portion of the noncoding repetitive DNA with each cell division, and this shortening of telomeric DNA is a major mechanism leading to cellular senescence after multiple rounds of cell division [17]. Recent studies have suggested that the preservation of telomere length and replicative capacity is positively correlated with the engraftment efficiency and antitumor efficacy of T-cell lines adoptively transferred into patients with melanoma [11]. Consequently, for clinical purposes, one potential strategy to expand the lifespan of CAR T cells is to develop a safe method to preserve the length of telomeres in these cells.

In recent years, synthetic mRNAs have been used to express ectopic genes, which has obvious advantages over traditional DNA-based methods [18, 19]. In contrast to constitutive overexpression using DNA vectors, genes encoding modified mRNAs do not integrate into the genome, leading to the transient expression of ectopic genes in cells [19]. Furthermore, unlike DNA vectors that must be transfected into the nuclei of cells for ectopic gene expression, mRNAs only require transfection into the cellular cytoplasm to achieve protein expression. Therefore, this method can be applied to the expression of ectopic genes in a broad range of cell types, including cell types that are typically difficult to transfect. Notably, recent advances in the modification of synthetic mRNAs have greatly reduced the cellular innate immune response triggered by mRNA delivery [20], thereby advancing the application of mRNA delivery in ectopic gene expression. Thus, this method has been used to express different genes in multiple cell types [20–24]. Accordingly, this method could also be used to transiently elevate telomerase activity in CAR T cells and solve the associated safety problems in clinical applications.

The aim of the present study was to solve the problem of the limited lifespan of CAR T cells through the transient delivery of modified telomerase reverse transcriptase (TERT) mRNA into CD19 CAR T cells. The results showed that the delivery of modified mRNA encoding hTERT to human CAR T cells improved the persistence and antitumor effects of these cells in mouse xenograft tumor models of B-cell malignancies compared with conventional CAR T cells.

## Results

### Generation of third-generation costimulatory CD19 CAR-modified T cells with *in vivo* antitumor activity

We designed a third-generation costimulatory CD19 CAR, harboring a combination of CD3ζ, CD28 and 4-1BB activation domains (Supplementary Figure S1A). To achieve the high expression of CD19 CAR in human T cells, an EF1α promoter was used to drive the expression of CD19 CAR. The expression of CD19 CAR was robustly detected after transduction into human T cells (Supplementary Figure S1B and C). CD19 CAR-transduced T cells were further expanded *in vitro* using IL-2. The starting cell number was about 10^7^, and whole T cells were increased to more than 10^9^ cells (>100-fold expansion) after 2 weeks of expansion *in vitro*, and 29.7% of the expanded T cells were CD19 CAR-positive cells (Supplementary Figure S1D). When co-cultured with CD19^+^ human Raji Burkitt’s lymphoma tumor cells at different E:T ratios (1:1, 10:1, 25:1), the expanded CD19 CAR T cells specifically secreted significantly higher levels of immunological cytokines compared with cells co-cultured with CD19^−^ K562 cells (Supplementary Figure S1E). Furthermore, CD19 CAR T cells efficiently killed CD19^+^ Raji cells but not CD19^−^ K562 cells (Supplementary Figure S1F).

**Figure 1:**
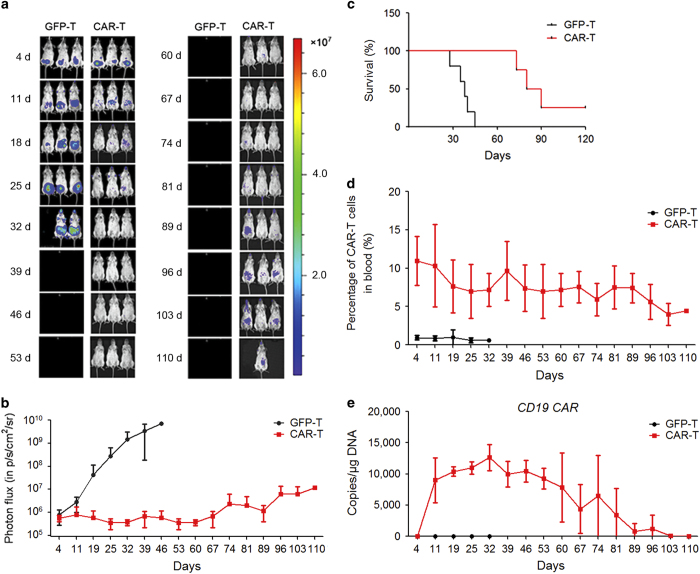
CD19 CAR T cells display significant antitumor activity in an immunodeficient mouse model of CD19^+^ human Burkitt’s lymphoma cells. (**a**) Weekly IVIS images of mice to assess the Raji (stably expressing the firefly luciferase fusion protein, Fluc) tumor burden after treatment (GFP-T: *n*=6, CAR-T: *n*=6). Raji cells (5×10^4^) expressing the firefly luciferase fusion protein (Fluc) were inoculated i.p. and after 4 days the mice were administered a dose of CAR T cells or GFP T cells (1×10^6^) by i.p. injection. Pseudocolor images show the light intensity and anatomic localization of the Fluc-derived Raji flux signals in three representative mice. The color bar displays the relative Fluc activity in p s^−1^ cm^-2^ sr^−1^. (**b**) Longitudinal monitoring of the bioluminescent signals of Fluc^+^ Raji cells in NPG/Vst mice. The *y* axis indicates the photon flux (p s^−1^ cm^-2^ sr^−1^). (**c**) Kaplan–Meier survival curve for NPG/Vst mice inoculated with Raji tumor cells after treatment with different T cells. Survival curves for the indicated CAR T cell groups were compared using the log-rank test. The CAR T group shows a significantly increased median survival (log-rank test, *P*<0.01) compared with the GFP T group. (**d**, **e**) Persistence and proliferation of CAR-modified T cells *in vivo*. Detection of CAR T cells in the blood of Raji-inoculated NPG/Vst mice after weekly T-cell injection using qPCR and flow cytometry. A total of 100 μl of venous blood was drawn weekly from orbital venous plexus of each mouse, and 50 μl of venous blood was lysed using FACS Lysing Solution. The CD19 CAR T cells were detected using a CD19 CAR-specific monoclonal antibody and flow cytometry. Another 50 μl aliquot of the venous blood was used to detect the copy numbers of CAR per microgram genomic DNA using qPCR.

To investigate the antitumor activity of CD19 CAR-modified T cells *in vivo*, a xenogeneic tumor model was established. Immunodeficient NPG/Vst mice were inoculated with CD19^+^ Raji cells expressing firefly fluorescent luciferase fusion protein. Subsequently, human T cells transduced with either CD19 CAR or GFP lentiviral vectors were inoculated into the mouse model. After adoptive immunotherapy, the tumor growth in the mouse models was weekly assessed using a bioluminescent (IVIS) imaging system (Figure 1a and b). Compared with GFP-transduced T cells, CD19 CAR T cells effectively inhibited tumor growth in mice (Figure 1a and b). Furthermore, while all the mice in the control group died before 40 days, most of the mice in the CD19 CAR T group survived for more than 70 days (Figure 1c), suggesting that CD19 CAR T-cell immunotherapy could increase the survival rate of mice with CD19^+^ tumor cells. However, the survival rate of mice in the CD19 CAR T group was gradually decreased beyond 70 days (Figure 1c). Histologic analysis of tissue sections of sacrificed mice indicated tumor infiltration in the kidney (Supplementary Figure S2A and B), the lung (Supplementary Figure S2C and D) and the liver (Supplementary Figure S2E and F), which eventually led to the death of the mice. To determine whether this effect reflected the reduction of CD19 CAR T cells *in vivo*, we further examined the number of CAR T cells in these mice. Indeed, although a high level of CAR T cells was maintained during the first 60 days, this number gradually decreased beyond 60 days (Figure 1d). Similar results were obtained when evaluating the copy number of CD19 CAR in CD19 CAR T cells in these mice (Figure 1e). Taken together, these results indicate that although third-generation costimulatory CD19 CAR-modified T cells efficiently eliminated CD19^+^ tumor cells *in vivo*, the limited lifespan significantly limited the therapeutic effects of CAR T cells.

### The synthesis and delivery of modified TERT mRNA transiently increased telomerase activity and extended telomere length in CAR T cells

**Figure 2:**
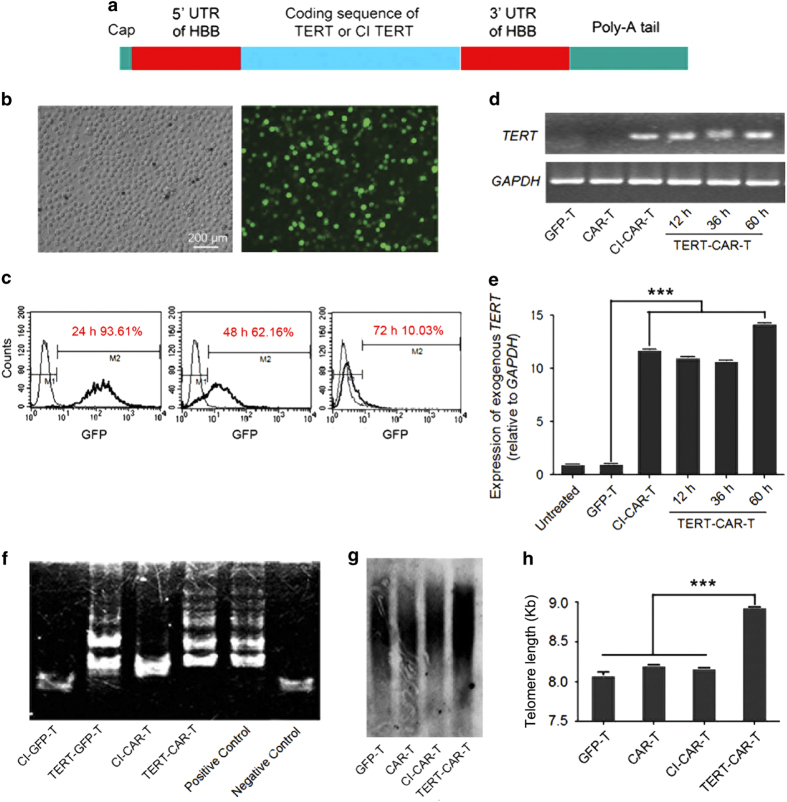
Synthesis and delivery of modified TERT mRNA transiently increased telomerase activity and extended telomeres length in CAR T cells. (**a**) Schematic of modified mRNA comprising the coding sequence of the full-length functional form of TERT or a CI form of TERT, flanked by HBB UTRs and a 151 nt poly-A tail, synthesized using modified nucleotides pseudouridine and 5-methylcytidine. (**b**) Transfection efficiency of T cells treated with modified mRNA encoding GFP at 12 h post transfection. (**c**) The transfection efficiency of T cells treated with modified mRNA encoding GFP measured using flow cytometry at 24 h post transfection exceeded 93.61%, and 62.6% of the cells were GFP positive after 48 h, whereas only 10% remained after 72 h. (**d**,**e**) The levels of hTERT mRNA were measured using RT-PCR and RT-qPCR. Treatment of CAR T cells with the same concentration of exogenous TERT mRNA or CI-TERT mRNA resulted in internalization of similar amounts of mRNA. (**f**) Detection of telomerase activity in T cells transfected with modified TERT mRNA using the TRAP. Telomerase activity indicated using a gel-based TRAP assay was detected in T cells treated with 2.0 μg TERT mRNA but not in controls, including cells treated with up to 2.0 μg of CI-TERT mRNA. White bands at the bottom of the gels represent positive controls for the PCR reaction. The sporadic bands in the vehicle lanes were artifacts due to primer dimers. (**g**, **h**) Telomere length was extended following modified TERT mRNA delivery. T cells were treated with 2.0 μg of TERT mRNA or CI-TERT mRNA three times every 24 h. Cells were collected for telomere length measurement at 7–10 day, and replicates were used for growth curve measurement, and in some cases, additional treatment at a later time point. *P<0.05, ***P<0.001.

To extend the lifespan of CD19 CAR-modified T cells, we overexpressed TERT, a major component of telomerase, in CD19 CAR T cells, as recent studies have suggested that the preservation of telomere length is positively correlated with the antitumor efficacy of adoptively transferred T cells. To avoid the potential genomic instability resulting from constitutive TERT overexpression, we designed a modified TERT mRNA (TERT mmRNA) (Figure 2a) that could not integrate into the genome of transduced cells and gradually degrades during cell division. A control mRNA vector was also constructed, encoding a catalytically inactive (CI) form of TERT. The TERT and CI-TERT mmRNAs were transfected into T cells by electroporation. To evaluate the transfection efficiency, GFP mmRNA was also electroporated as a control group (Figure 2b), and a high transfection efficiency (93.1±2.0%) was achieved 24 h after electroporation. In addition, ~85–90% of T cells survived after electroporation (data not shown). Next, the presence of a high level of TERT mmRNA in transduced human T cells was confirmed (Figure 2c–e). The expression of exogenous TERT mmRNA at different time points were also checked by reverse transcription (RT)-PCR, which showed that it peaked at 24 h after electroporation and decreased to baseline levels within 72 h (Supplementary Figure S3A).

To examine whether TERT mmRNA transfection resulted in the production of functional TERT protein, we evaluated the telomerase activity in TERT mmRNA-transduced T cells. Notably, telomerase activity was detected in either conventional T cells or CD19 CAR T cells transduced with TERT mmRNA but not in T cells transduced with GFP or CI-TERT mmRNA (Figure 2f). Moreover, the telomere length was significantly increased in TERT mmRNA-transduced CAR T cells (Figure 2g and h) but not in CI-TERT mmRNA-transduced CAR T cells. Therefore, these results suggest that the transient expression of TERT mRNA efficiently enhanced telomerase activity and telomere length in CAR T cells.

### TERT mmRNA delivery significantly enhanced proliferation and inhibited cell senescence in CD19 CAR T cells

**Figure 3:**
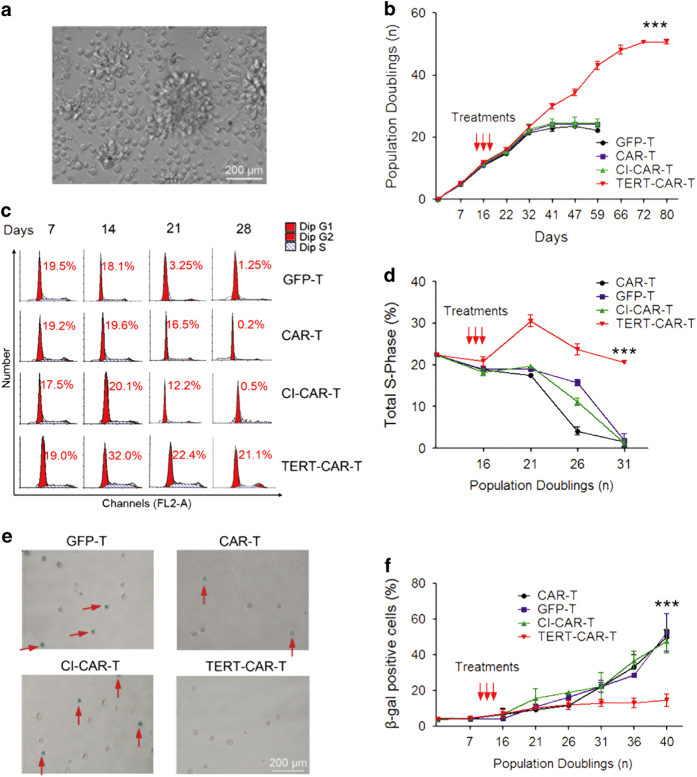
Modified TERT mRNA delivery to T cells increased proliferative capacity and transient reduction of senescence-associated markers. (**a**) The clones of CD19 CAR T cells 7 days post transfection with TERT mRNA. (**b**) Growth curves of CD19 CAR T cells transfected with TERT mRNA, CI-TERT mRNA, three times in succession at 24 h intervals starting at PD 13, and the growth curves were repeated twice with each population cultured in triplicate compared with untreated CAR T cells. *P<0.05, ***P<0.001. (**c**) The cell cycle analysis was performed using flow cytometry (TERT mRNA group, CI-TERT mRNA group, GFP T group or CAR T group). (**d**) Only the percentage of total S-phase of each T cell line was statistically summarized. The percentage of total S-phase represents the proliferation of T cells. The data are presented as the mean±s.d. of results from three independent experiments. *P<0.05, ***P<0.001. (**e**, **f**) Quantification of senescent marker expressing T cells after modified TERT mRNA transfection three times in succession at 24 h intervals. The β-gal-positive T cells are shown in grayish-green in the microscope images. Before β-gal staining, the T cells were collected using centrifugation, washed with PBS and resuspended. The data are presented as the mean±s.d. of results from three independent experiments. *P<0.05, ***P<0.001

To determine whether TERT mmRNA delivery promoted the proliferation of CD19 CAR T cells, the number of CAR T cells was calculated at different time points during *in vitro* expansion (Figure 3a). Untreated and CI-TERT mmRNA-transduced CAR T cells gradually stopped proliferating after ~20–25 population doublings (PDs) (~6 weeks), whereas cells transduced with TERT mmRNA three times in succession continued to proliferate for an additional 15 PDs (4 weeks; Figure 3b). In the long-term culture, the telomere length in TERT mmRNA-transduced CAR T cells gradually declined until the cells stopped dividing (Supplementary Figure S3B). As the starting cell number was about 1×10^6^ after mmRNA delivery, the whole T cells of TERT mmRNA-transduced was increased to 3.0±0.22×10^8^ (300-fold expansion), but the whole cell number of either untreated CAR T cells or CI-TERT mmRNA-transduced was about 3.7±0.75×10^7^ (37-fold expansion). We further examined the percentage of T cells at S-phase at different time points during expansion (Figure 3c) as an indicator of the proliferation rate. Consistent with an increase in the total cell number, CD19 CAR T cells transduced with TERT mmRNA maintained a relative high percentage of cells at S-phase (~20%), but the percentage of control cells at S-phase gradually decreased during expansion (Figure 3d). Using β-galactosidase (β-gal) staining to detect senescent cells, we further examined the extent of senescence in T cells under different treatments after *in vitro* expansion (Figure 3e). While the percentage of β-gal-positive cells was significantly increased in the control groups after 40 days of *in vitro* culture, the percentage of β-gal-positive CAR T cells transduced with TERT mmRNA was only slightly increased (Figure 3f). Taken together, these data suggest that TERT mmRNA delivery significantly enhanced proliferation and inhibited cell senescence in CD19 CAR T cells.

### TERT mmRNA delivery did not alter the phenotypic and functional characteristics of CAR T cells

**Figure 4:**
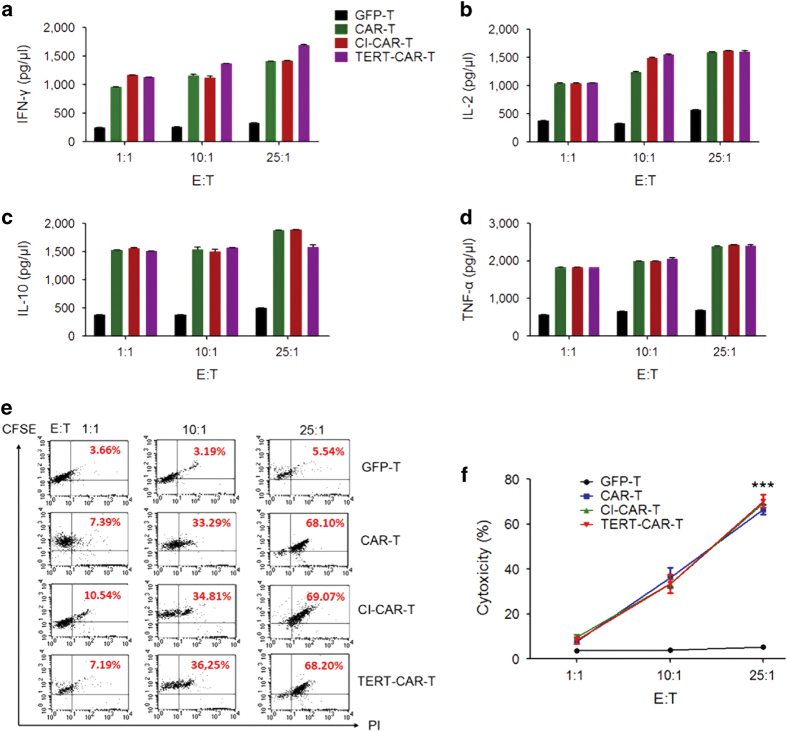
*In vitro* bioimmunity of TERT mRNA delivery CD19 CAR-modified T cells. (**a**–**d**) Cytokine release of TERT mRNA delivery to CD19 CAR-modified T cells was detected by enzyme-linked immunosorbent assay after co-culture with CD19^+^ tumor cells at different E/T ratio (1:1, 10:1, 25:1), and four types of cytokines (IL-2, IL-10, IFN-γ and TNF-α) were detected. The data are presented as the mean±s.d. of results from three independent experiments. *P<0.05, ***P<0.001. (**e**) CD19-specific CTL of TERT mRNA delivery to CD19 CAR-modified T cells by flow cytometry. Raji cells are CD19^+^ human Burkitt’s lymphoma cells, and K562 cells are CD19^−^ human lymphoma cells. (**f**) Cytolytic activity was determined after 4 h of co-culture. The data are presented as the mean±s.d. of results from three independent experiments. *P<0.05, ***P<0.001.

Next, we examined whether TERT mmRNA delivery affected the phenotype of CD19 CAR T cells. No obvious differences were observed in the expression of major T cell surface markers between the original CAR and TERT-CAR T cells (data not shown). Furthermore, CD19 CAR T cells transduced with TERT mmRNA showed a normal karyotype after 50 days of *in vitro* culture (data not shown). We next investigated whether TERT mmRNA delivery influenced the functions of CAR T cells. Four groups of T cells under different treatments (GFP-T, CAR-T, CI-CAR-T and TERT-CAR-T) were individually co-cultured with CD19^+^ Raji cells at different E:T ratios. CD19 CAR T cells transduced with TERT mmRNA showed levels of cytokine secretion comparable to the control groups (CAR-T and CI-CAR-T; Figure 4a–d). Moreover, there was no difference in CD19^+^ tumor killing efficacy among TERT-CAR-T, CD19 CAR-T and CI-CAR T cells (Figure 4e and f). CD19^−^ K562 cell line was also studied as a negative control for cytokine production and cytotoxic T lymphocytes (CTL) assay (Supplementary Figure S4A–F). Thus, these results suggest that TERT mmRNA delivery did not alter the phenotypic and functional characteristics of CAR T cells.

### Improved *in vivo* proliferation and persistence and enhanced antitumor efficacy of TERT-CD19-CAR T cells after adoptive transfer

**Figure 5:**
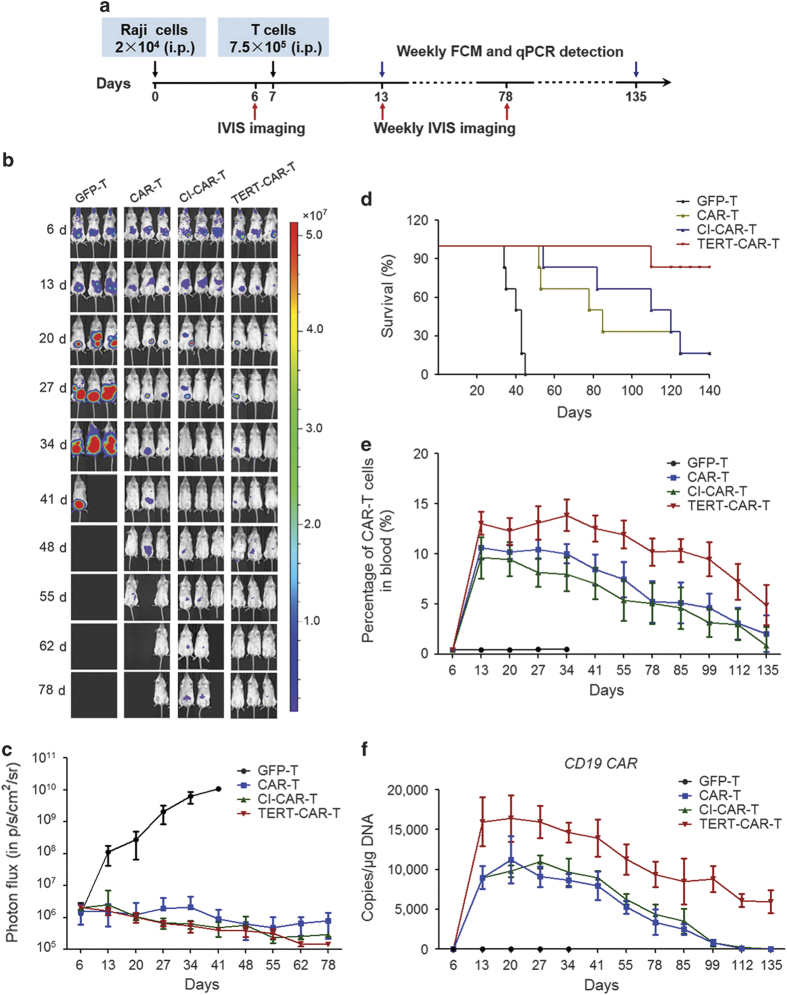
Long-term *in vivo* antitumor activity of TERT mRNA delivered to CAR T cells. (**a**) Schema of *in vivo* study demonstrating the antitumor activity of transduced T cells in a disseminated human B-cell malignancy xenogeneic NPG/Vst mice model (GFP-T: *n*=6, CAR-T: *n*=6, CI-CAR-T: *n*=6, TERT-CAR-T: *n*=6). (**b**) Antitumor activity of TERT mRNA delivery CAR T cells in NPG/Vst mice by weekly *in vivo* bioluminescent imaging. (**c**) Summary of the bioluminescence signal (relative Fluc activity in p s^−1^ cm^-2^ sr^−1^) as a measurement of tumor growth after tumor cell infusion. The *y* axis indicates the photon flux (p s^−1^ cm^-2^ sr^−1^). (**d**) Kaplan–Meier survival curves of mice receiving TERT mRNA delivery CAR T cell treatment, CI-TERT mRNA (CI) delivery CAR T cell treatment, untreated CAR T cells treatment or GFP T cells treatment. The survival curves for the indicated TERT mRNA delivery CAR T cell groups were compared using the log-rank test, showing a significantly increased median survival compared with the CAR T group, and when comparisons were made between the TERT-CAR T group and CI-CAR T group, the data exhibited significant difference (*P*=0.0002, *P*<0.01) (log-rank test, *P*<0.01). (**e**, **f**) Persistence and proliferation of CAR T cells *in vivo*. Detection of CAR T cells in the blood of Raji-inoculated NPG/Vst mice every week after T-cell injection using flow cytometry and qPCR. When comparisons were made between the TERT-CAR T group and CI-CAR T group, the data exhibited significant difference in **e** *P*=0.0013 (*P*<0.01), and in **f** *P*=0.0117 (*P*<0.05).

To evaluate the antitumor efficacy of TERT-CD19-CAR T cells *in vivo*, we co-injected TERT-CAR T cells into mouse models with CD19^+^ Raji cells (Figure 5a). Raji tumor progression was monitored weekly through bioluminescent (IVIS) imaging after the injection of T cells under different treatments (Figure 5b and c). The initial *in vivo* antitumor efficacy of TERT-CAR T cells was similar to that of CI-CAR-T or untreated CAR T cells (Figure 5b and c). However, the mice in the untreated CAR T group started dying after 55 days. In contrast, mice in the TERT-CAR T group remained viable (Figure 5b). After 130 days of treatment, most of the mice from the control group died, but ~80% of mice from TERT-CAR T group remained alive (Figure 5d). Consistent with this observation, the total cell number of CAR T cells in the mice from TERT-CAR T group was higher than that in the mice from the control group (CAR-T and CI-CAR-T) after 130 days of treatment (Figure 5e). Similar results were obtained when evaluating the copy number of CD19 CAR in CD19 CAR T cells in these mice (Figure 5f). Taken together, these data indicate that CD19 CAR T cells transduced with TERT mmRNA have improved *in vivo* persistence and proliferation, thereby enhancing the long-term antitumor effects of CAR T cells *in vivo*.

### Adoptively transferred TERT-CAR T cells were genetically stable *in vivo*

**Figure 6:**
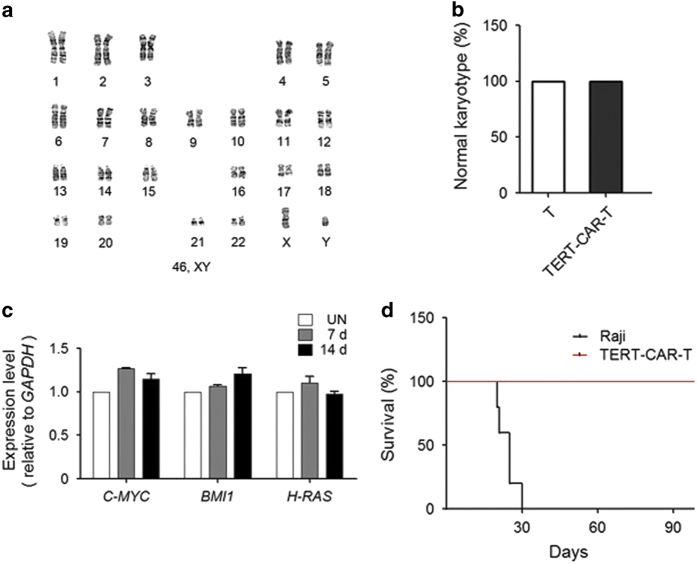
Safety of TERT mRNA delivery to CD19 CAR T cells. (**a**) The karyotypes of CD19-CAR T cells were analyzed using conventional Giemsa staining and G-banding with trypsin and Giemsa (GTG) at 14 days after TERT mRNA delivery. A representative karyotype for one of the TERT-CD19-CAR T cells showed a normal 46, XY chromosome count with no abnormalities. (**b**) Karyotype analysis performed on overall TERT-CD19-CAR T cells revealed a normal karyotypes. (Untreated T cell: *n*=8, TERT-CD19-CAR-T: *n*=8). (**c**) The expression of cancer genes *C-MYC, BMI1* and *H-RAS*, was detected by RT-qPCR relative to *GAPDH* at 7 and 14 days after TERT mRNA delivery. Untreated T cells (UN) were detected as control. (**d**) TERT-CD19-CAR T cells were subcutaneously injected into nude mice, and human Burkitt’s lymphoma Raji cells were subcutaneously injected as a positive control. Solid tumors formed after Raji cells were transplanted, and these mice remained alive for no more than 30 days and the mice in TERT-CD19-CAR T cell group were all in good condition.

Finally, we examined whether transient delivery of TERT mmRNA into CAR T cells may induce their genetic instability. After 14 days culture post transfection, TERT-CAR T cells still maintained normal karyotype (Figure 6a and b). Consistent with this result, the expression of key oncogenic genes *C-MYC*, *BMI1* and *H-RAS* in these TERT-CAR T cells did not show significant change at different time points after mmRNA transfection (Figure 6c). Furthermore, mice transplanted with only TERT-CAR T cells were 100% viable with no tumor formation after 90 days of injection (Figure 6d). Therefore, these results suggested that adoptively transferred TERT-CAR T cells are genetically stable *in vivo*.

## Discussion

In the present study, we developed a novel approach to enhance the lifespan of CAR T cells through the transient delivery of a modified mmRNA encoding TERT. After long-term culture *in vitro*, CAR T cells transduced with TERT mmRNA showed increased telomerase activity, extended telomeres, enhanced proliferation and delayed cellular senescence compared with untreated controls. More importantly, when injected into the mouse xenograft tumor model of human B-cell malignancy, these modified CD19 CAR T cells exhibited not only enhanced *in vivo* persistence and proliferation but also showed improved long-term antitumor efficacy *in vivo*.

Although *in vitro* expansion of CAR T cells is required to generate sufficient cell numbers for killing tumor cells *in vivo*, the prolonged *in vitro* culture of CAR T cells might lead to senescence, thereby reducing the long-term persistence of these cells *in vivo*. Indeed, third-generation CAR T cells with integrated activation domains required for T cell proliferation showed decreased cell numbers beyond 60 days post injection in the mouse model (Figure 1d and e), and the long-term survival of mice treated with CAR T cells also significantly decreased (Figure 1c). In contrast, CAR T cells transiently transduced with TERT mmRNA showed enhanced long-term persistence *in vivo* (Figure 5e and f), and the survival rate of mice treated with TERT-CAR T cells remained high at 140 days post injection (Figure 5c). These results were consistent with previous observations that the clinical efficacy of CAR T-cell immunotherapy is primarily dependent on the *in vivo* persistence of CAR T cells [8, 12, 25, 26]. More importantly, the enhancement of the survival rate of the mice after TERT-CAR T cell injection also indicated that the long-term antitumor efficacy of CAR T cells could be enhanced by the transient delivery of TERT mmRNA. Furthermore, this transient delivery also markedly enhanced the proliferation of CAR T cells during *in vitro* culture (Figure 3a). Therefore, the results of the present study provide a simple and robust method to generate large amounts of CAR T cells with long-term antitumor efficacy.

One prominent advantage of using TERT mRNA delivery to extend telomere length in CAR T cells is that this method avoids potential safety problems, which is a major concern in the clinical application of CAR T-cell immunotherapy. Previous studies attempting to enhance T-cell lifespan through TERT overexpression primarily involved the constitutive overexpression of TERT [17, 27–29]. However, it has been reported that the genomic integration and constitutive expression of the TERT transgene resulted in chromosome instability [11, 28–30], causing safety problems in clinical applications. In contrast to previous studies, we used modified mRNAs that do not integrate into the genome of recipient cells, thus avoiding potential genomic instability [20–24]. Indeed, in the present study, CAR T cells modified using this approach showed normal karyotypes, phenotypes and T-cells functions, and cell proliferation remained dependent on cytokine or antigen stimulation (Figures 4 and 6). Furthermore, these data also demonstrated that the transient delivery of TERT mRNA into CAR T cells during *in vitro* culture was sufficient to extend the telomere length (Figure 3), which significantly enhanced the *in vivo* antitumor efficacy of these cells (Figure 5). Accordingly, the superior features of mRNA delivery show the potential use of this approach in clinical applications of CAR T cells.

In summary, these data demonstrate that the transient delivery of modified mRNA encoding TERT enhances persistence and proliferation both *in vitro* and *in vivo* and improves the *in vivo* antitumor efficacy of CAR T cells. This new approach provides an effective and safe method to improve the therapeutic potential of CAR T cells. Although the clinical application of second- or third-generation CAR T cells for the treatment of B-cell malignancies has made significant advances in recent years [31–34], progress to apply current CAR T-cell immunotherapy for the treatment of other types of cancer, particularly solid tumors, remains limited, primarily reflecting the insufficient persistence and proliferation of CAR T cells *in vivo* [35–37]. Thus, the approach developed in the present has great potential for treating other types of cancers, particularly solid tumors, in future studies.

## Materials and Methods

### Culture medium

T cells were cultured in T-cell medium comprising X-VIVO 15 medium (Lonza, Basel, CH, Switzerland) supplemented with 5% fetal bovine serum (FBS) (Gibco, LAX, CA, USA), 100 U ml^−1^ penicillin, 100 μg ml^−1^ streptomycin, 1.25 μg ml^−1^ amphotericin B, 2 mM L-glutamine (Gibco), and 100 U ml^−1^ hIL-2 (PerproTech, Rocky Hill, CT, USA). The T cells were activated using CD3- and CD28-specific magnetic beads at three beads/cell (Invitrogen Life Technologies, Carlsbad, CA, USA). Raji and K562 cells were cultured in R10 medium comprising Roswell Park Memorial Institute 1640 (Hyclone, Logan, UT, USA) supplemented with 10% FBS, 100 U ml^−1^ penicillin, 100 μg ml^−1^ streptomycin (Gibco) and 2 mM L-glutamine (Gibco). In all, 293T cells were cultured in D10 medium comprising Dulbecco’s modified Eagle’s medium (Hyclone, UT, USA) supplemented with 10% FBS, 100 U ml^−1^ penicillin, 100 μg ml^−1^ streptomycin, and 2 mM L-glutamine. The cytotoxicity medium contained phenol red-free Roswell Park Memorial Institute supplemented with 5% FBS, 100 U ml^−1^ penicillin, and 100 μg ml^−1^ streptomycin.

### Anti-CD19 CAR lentiviral vector design and generation

A third-generation anti-CD19 CAR recombinant lentiviral vector was designed and referred to as pRRL-EF1A-19CAR3. It included the following components from 5′ to 3′: the VSVG lentiviral backbone, the FMC63 scFv, the hinge and transmembrane regions of the CD8 molecule, the cytoplasmic portions of CD28 and 4-1BB, and the cytoplasmic component of the CD3-ζ molecule. This vector was constructed using a multistep strategy that bridged fragments encoding the CD8, CD28, 4-1BB and CD3-ζ components. The vector backbone (pRRLSIN.cPPT.PGK-GFP.WPRE) was a gift from Didier Trono (Addgene plasmid, NO. 12252, MA, USA). This vector was modified with the EF1A promoter, inserted between BstXI and XbaI sites. FMC63-28Z encoding the anti-CD19 CAR (FMC63) was obtained from GenBank (NO. HM852952). The sequences for FMC63 ScFv were synthesized at OriGene Technologies (Beijing, China). Anti-CD19 CAR includes ScFv, the transmembrane and intracellular domain of human CD28, and the activation domain of 41-BB and the cytosolic signaling domains of CD3ζ. These domains were linked using over-extension PCR and cloned into the backbone vector via XbaI sites. CAR expression was easily detected by correlation with enhanced green fluorescent protein (EGFP) fluorescence using P2A bicistronic expression. The new lentiviral vector was named pRRL-EF1A-19CAR3. A plasmid encoding the GFP protein was cloned into the lentiviral vector with a portion of the *CD28* gene and the gene for the cytoplasmic portion of the CD3ζ molecule to form the GFP-28Z plasmid as a negative control.

### CD19 CAR lentivirus production

To produce perpetual lentivirus particles, 293T-packaging cells were transfected with the pMD2.G plasmid, psPAX plasmid and the vector plasmid using Lipofectamine 2000 reagent (Invitrogen Life Technologies). The transfected cells were incubated at 37 °C for 6–8 h in D10 medium without antibiotics. The medium used for transfection was subsequently replaced with fresh D10 medium, and the cells were incubated for another 24–48 h. During and after transfection, the 293T cells were cultured in 10 cm dishes. Supernatant containing the lentivirus particles was collected and filtered through a 45-μm filter to remove cellular debris. The supernatant was concentrated by ultracentrifugation at 20 000 *g* for 90 min at 4 °C. The viral pellet was resuspended in phosphate-buffered saline (PBS) overnight at 4 °C. The re-supernatant was snap frozen on dry ice and stored at −80 °C. The viral titers were determined after the infection of active human T cells at a multiplicity of infection of 10–20 in the presence of 10 mg ml^−1^ Polybrene (Sigma, St Louis, MO, USA).

### *Ex vivo* costimulation and the massive proliferation of T cells

Human peripheral blood mononuclear cells were isolated by density gradient centrifugation over Ficoll-Paque-Plus (Pharmacia Biotech, Piscataway, NJ, USA) and activated using CD3/28 beads at a concentration of three beads/cell in 5% FBS-X-VIVO 15 media containing 100 U ml^−1^ IL-2 at 37 °C and 5% CO_2_ for 1–2 days. After 1–2 days costimulation, the T cells among the peripheral blood mononuclear cells were specifically activated. Subsequently, the T cells were added to culture flasks pre-treated with RetroNectin (Takara, Tokyo, Japan) and loaded with the lentiviral anti-CD19 CAR vector at an multiplicity of infection of 10–20. The cells were transfected and cultured at 37 °C and 5% CO_2_ in X-VIVO 15 media for 24 h in the presence of 10 mg ml^−1^ Polybrene. The transfected T cells were cultured in fresh culture medium for at 37 °C and 5% CO_2_ and collected 14 days after culture.

### Flow cytometry

The cell surface phenotype of genetically modified T cells and tumor lines was determined using flow cytometry. A total of 10^5^–10^6^ T cells were collected 72 h after co-culture with CD19 CAR or GFP lentivirus particles, washed with PBS supplemented with 200 μl of staining buffer and subsequently treated with fluorescein isothiocyanate-conjugated, phycoerythrin-conjugated or TRITC-conjugated mAbs specific for T-cell receptor α/β (TCRαβ), CD3, CD4 and CD8 (BD Biosciences, San Jose, CA, USA). The tumor cells were determined after staining with the CD19-specific mAb (BD Biosciences). CD19-CAR was detected using Biotin-SP-AffiniPure F(ab’)2 Fragment Goat Anti-Mouse IgG (Jackson, Lancaster, PA, USA) and the SAV-APC antibody (BD Biosciences). After 30–60 min incubation on ice, the cells were washed twice and resuspended in PBS, followed by analysis on a FACSCalibur flow cytometer (BD Immunocytometry Systems, San Jose, CA). CellQuest software (BD Immunocytometry Systems) was used to calculate the percentage of cells and mean fluorescent intensity within a given region of the histogram.

To measure the cell cycle phase, the cells were collected, washed thrice with ice-cold PBS, and fixed with 70% ice-cold ethanol overnight. Before analysis, the cells were stained with propidium iodide (PI, 50 μg ml^−1^) (Sigma) in the presence of RNase (100 μg ml^−1^). After no more than 30 min, the cells were collected and analyzed by flow cytometry to detect the cell cycle phase.

To measure the CAR T cells in blood, a total of 100 μl venous blood was drawn weekly from orbital venous plexus of each mouse into 0.5 ml K_2_EDTA anticoagulation centrifuge tubes and 50 μl of venous blood was stained with Biotin-SP-AffiniPure F(ab’)2 Fragment Goat Anti-Mouse IgG (Jackson) and the SAV-APC antibody (BD Biosciences) and isotype was stained with Biotin-SP-AffiniPure Goat Anti-Mouse IgG and the SAV-APC antibody. After 30–60 min incubation on ice, whole blood samples were lysed using (1×) BD FACS lysing solution and then the cells were washed twice and resuspended in PBS, followed by analysis on a FACSCalibur flow cytometer (BD Immunocytometry Systems). CellQuest software (BD Immunocytometry Systems) was used to calculate the percentage of cells and mean fluorescent intensity within a given region of the histogram.

### Cytokine production

Duplicate wells containing 10^6^ genetically modified CD8^+^ T cells were co-incubated with 10^6^ stimulator cells irradiated with 8 000 cGy in 2 ml of culture media for 72 h in the presence of rhIL-2 at 5 U ml^−1^. The cell-free supernatants were collected and assayed for cytokine content using an enzyme-linked immunosorbent assay (R&D Systems, Minneapolis, MN, USA), and the concentration was extrapolated from a standard curve.

### Flow-cytometric CTL assay

As target cells, a CD19^+^ human lymphoma cell line (Raji cells) and a CD19^−^ human lymphoma cell line (K562 cells) was suspended in PBS+0.1% bovine serum albumin at 1×10^6^ cells per ml. The fluorescent dye carboxyfluorescein diacetate succinimidyl ester (CFSE; Invitrogen Life Technologies) was used to stain target cells at a concentration of 2.5 μM. After incubation, the labeling reaction was terminated after the addition of FBS at a volume equal to that of the cell suspension, and the cells were incubated for 2 min at room temperature. The cells were washed and suspended in cytotoxicity medium. Effector T cells were washed and suspended at 5×10^6^ cells per ml in cytotoxicity medium. In all experiments, the cytotoxicity of effector T cells transduced with the anti-CD19 CAR was compared with the cytotoxicity of negative control effector T cells. CD19 CAR-transduced effector T cells and negative control effector T cells were cultured in a 96-well culture plate in duplicate at T cell:target cell ratios of 25:1, 10:1 and 1:1. Each culture system also contained CD19-negative K562 cells as a control. A total of 10^4^ CD19-positive target cells and 10^4^ CD19-negative control cells were used in each test. The culture systems were incubated for 4 h at 37 °C. Immediately after the incubation, 50 μg ml^−1^ PI (propidium iodide) was added to stain dead cells, and flow cytometry was performed using a BD FacsCanto II flow cytometer (BD Biosciences). Analysis was gated on CFSE-positive cells, and the percentages of live target cells and live CD19-negative control cells were determined for each T cell and target cell culture. For each T cell and target cell culture, the corrected percent survival of CD19-positive target cells was determined by dividing the percent live CD19-negative cells in each T cell and target cell culture by the ratio of the percent CD19-positive target cells: percent CD19-negative control cells in tubes containing only CD19-positive target cells and CD19-negative control cells without any effector T cells. This correction accounted for variations in the starting cell numbers and spontaneous target cell death. Cytotoxicity was calculated as 100% (corrected percent survival of CD19-positive target cells). For all E:T ratios, the cytotoxicity was determined in duplicate, and the results were averaged. The percentage of killing of target cells was determined as: Ratio=percentage of CFSE^+^PI^+^ target cells/percentage of CFSE^+^ target cells×100%.

### *In vivo* bioluminescence of tumors

Severe combined immune deficiency NPG/Vst mice obtained from the VITALSTART, were used as the mouse xenograft model. NPG/Vst mice are members of the NOD-Prkdc^scid^ Il2rg^null^ family and have been internationally recognized as the highest immune deficiency mouse model and the most suitable tool for cell transplantation. At 6–12-weeks-old, female mice with severe immunodeficiency NPG/Vst were inoculated intraperitoneal (i.p.) with 5×10^4^ Raji human CD19^+^ Burkitt’s lymphoma cells expressing the firefly luciferase fusion protein (Luc). After 4 days, the mice were administered a dose of CD19 CAR T cells or GFP-transduced T cells by i.p. injection. Only mice that had equal tumor burden (1.5±0.5×10^5^ photons per sec) before T-cell injection were used. Mice with lesser or greater tumor burden were excluded from this study. Tumor-bearing mice retained in this study were randomized to different treatment groups (at least three mice per group). No blind method was used. The T-cell dose was based on the percentage of CAR^+^ cells measured by pre-injection CTL analysis. The tumor burden was monitored twice a week by *in vivo* bioluminescence imaging (IVIS 100 Imaging System). Living Image software version 4.3.1 (Caliper Life Science, Waltham, MA, USA) was used to acquire and quantify the bioluminescence imaging data sets. Before weekly IVIS bioluminescence imaging, the mice were infused by i.p. injection with 150 mg kg^−1^ D-luciferin (Xenogen, Jena, Germany) suspended in 200 μl PBS. After 10 min, the mice were imaged under 2% isoflurane anesthesia. Image acquisition was performed with a 15- or 25-cm field of view at medium binning level for 0.5 to 3 min exposure time. Both dorsal and ventral views were obtained for all animals. Tumor bulk was determined by IVIS imaging. The animal experiment protocol was approved by the Biomedical Research Ethics Committee of Peking University and strictly adhered to the American Physiological Society’s ‘Guiding Principles in the Care and Use of Vertebrate Animals in Research and Training’.

### Real-time PCR and quantitative real-time PCR

Total mRNA was extracted using TRIzol reagent (Invitrogen Life Technologies). Reverse transcription was performed using the Superscript III First-Strand Synthesis supermix for RT-PCR (Invitrogen Life Technologies) and RNA (0.1 μg) was reverse transcribed into cDNA. PCR was performed with Taq polymerase (Invitrogen Life Technologies) for 30 cycles (denaturation: at 95 °C for 30 s; annealing: at 58 °C for 30 s; extension: at 72 °C for 30 s). PCR-amplified product was subsequently size fractioned on 1% agarose gel, stained with ethidium bromide and visualizedunder ultraviolet light. qPCR for specific genes was performed using the respective probe-based TaqMan Gene Expression assays (Applied Biosystems). The reactions were performed in duplicate using an ABI PRISM 7500 Sequence Detection System (Applied Biosystems). The relative expression was calculated using the DDCt (2^−ΔΔCt^) method with *GAPDH* as an endogenous control.

After treatment, 50 μl of venous blood was extracted weekly from the orbital venous plexus of the mice into 0.5 ml K_2_EDTA anticoagulation centrifuge tubes (Beijing Emilion Science &Technology, Beijing, China). Genomic DNA was directly isolated from whole blood using the QIAamp DNA Blood Midi Kit (Qiagen, Hilden, Germany), quantified using a spectrophotometer, and stored at −80 °C. The qPCR analysis of the genomic DNA samples was performed in bulk using 100 ng of genomic DNA, ABI Taqman technology and a validated assay to detect the integrated CD19 CAR transgene sequence. Pass/fail parameter ranges, including standard curve slope and *r* ^2^ values, the accurate quantification of the reference sample (1 000 copies/plasmid spike) and the absence of the potential amplification of the DNA sample obtained from healthy mice were established and used to define pre-established acceptance ranges for assay performance. The primer/probes for the CD19 CAR transgene were designed as previously reported [38]. To determine the copy number/unit DNA, an eight-point standard curve was generated comprising 10^6^ to 5 copies lentivirus plasmid spiked into 100 ng non-transduced control genomic DNA. Each data point (samples, standard curve and reference samples) was evaluated in triplicate, and the average values were reported. The amplification reactions generated a correction factor (ng detected/ng input). The transgene copies/microgram DNA were calculated according to the following formula: copies calculated from *CD19* standard curve/input DNA (ng)×correction factor×1 000 ng. The accuracy of this assay was determined as the ability to quantify the marking of the infused cell product by qPCR according to the following formula: Average marking=detected copies/input DNA×6.3 pg DNA/male somatic cell×correction factor, versus transgene positivity by flow cytometry using CAR-specific detection reagents.

Primers were designed using Primer5 or from the articles of Ramunas *et al.* [23] and were as follows. *GAPDH*: 5′-GTCAAGGCTGAGAACGGGAA-3′ (Forward), 5′-AAATGAGCCCCAGCCTTCTC-3′ (Reverse); *CD19-CAR*: 5′-ACATCCTCCCTGTCTGC-3′ (Forwad1), 5′-ATCCTCCCTGTCTGCC-3′ (Forwad2), 5′-CCACTGCCACTGAACC-3′ (Reverse); *H-RAS*: 5′-CGGAAGCAGGTGGTC-3′ (Forwad), 5′′-TGGTGTTGTTGATGGC-3′ (Reverse); *C-MYC*: 5′-ACCCTTCTCCCTTCG-3′ (Forward), 5′-CCGCTCCACATACAGT-3′ (Reverse); *BMI1*: 5′-GCCACAACCATAATAGAA-3′ (Forward), 5′-ACTTGGACATCACAAATAG-3′ (Reverse); Exogenous TERT mRNA: 5′-GTCACCTACGTGCCACTCCT-3′ (Forward), 5′-AGCAAGAAAGCGAGCCAAT-3′ (Reverse).

### mRNA template generation and synthesis

To generate modified TERT mRNAs, the CI-TERT open reading frame (ORF) was inserted into the MCS of a starting plasmid containing the T7 promoter, the 5′ UTR of the human beta globin gene (*HBB*), the multiple cloning site (MCP), the 3′ UTR of *HBB*, and a 151 bp poly-A sequence, and a mutant was generated through a G516D modification in the ORF of the pBABE-neo-hTERT plasmid (Addgene plasmid 1774). Residue 516 is in the QFP (a motif associated with the multimerization and RNA binding of TERT) motif of the N-terminal extension of TERT, a motif associated with the multimerization and RNA binding of TERT, essential for TERT interaction with TERC RNA. The CI-TERT mutant was generated from the TERT sequence after introducing the D712A mutation. Linearization was achieved using a class II restriction enzyme according to the poly-A sequence. The resulting intermediate plasmid was sequenced, linearized and transcribed using RNA polymerase from the MEGAscript T7 Kit (Ambion, Austin, TX, USA) and a custom nucleotide mix of canonical and non-canonical nucleotides (TriLink BioTechnologies, San Diego, CA, USA) in which the final nucleotide concentrations per 40 μl *in vitro* transcription reaction were 7.5 mM for each of adenosine-5′-triphosphate, 5-methylcytidine-5′-triphosphate (m5C), and pseudouridine-5′-triphosphate (Ψ), 1.5 mM for guanosine-5′-triphosphate, and 6 mM for the cap analog (ARCA, NEB), at a molar ratio of adenosine-5′-triphosphate:m5C:Ψ:guanosine-5′-triphosphate:ARCA of 1:1:1:0.2:0.8. To further decrease the potential immunogenicity of the mRNA associated with the 5′-3 P-bearing fraction, the *in vitro* transcription products were treated with phosphatase (Antarctic Phosphatase, NEB). The size and integrity of the mRNA products were verified using denaturing agarose gel electrophoresis. The human TERT ORF used to generate the DNA templates for mRNA synthesis was identical to the National Center of Biotechnology Information (NCBI) human TERT transcript variant 1 reference sequence NM_198253.2, generated by a G516D modification in the ORF of the pBABE-neo-hTERT plasmid (Addgene plasmid 1774). Residue 516 is in the QFP motif of the N-terminal extension of TERT, a motif associated with the multimerization and RNA binding of TERT, essential for TERT interaction with TERC RNA. The CI-TERT mutation was generated in the TERT sequence by a D712A modification [23].

### Cell electroporation

Human Raji CD19^+^ Burkitt’s lymphoma cells were cultured in R10 media, and subsequently the cells were collected by centrifugation and re-suspension. A total of 2 μg of the pGL4.51 plasmid (luc2/CMV/Neo; Promega E1320, Madison, MI, USA) per 2×10^6^ cells was electrotransfected using the T-021 program with Amaxa Nucleofector II Technology (Lonza). After culture in R10 media in the presence of 800 μM G418 (Invitrogen, Carlsbad, CA, USA) for ~2 weeks, the Raji cells showed the stable expression of a firefly luciferase fusion protein. CAR-modified T cells were cultured in 5% FBS-X-VIVO 15 media. For transient expression in activated CAR-modified T cells, the cells were suspended with 1 μg of modified mmRNA in 100 μl of the human T-cell Nucleofector Kit (Lonza) and subsequently applied to the T-023 program equipped with Nucleofector Technology.

### PD time measurement by cell counting

To obtain lifespan curves and population doubling (PD) time, cell cultures were serially passaged until the end of their proliferative capacity *in vitro* and the PD levels were determined. PD time (t)=T/3.32(lgN2-lgN1) (where T is the duration of logarithmic phase of growth, N1 and N2 are the number of cells at the beginning and end of the logarithmic phase of growth) [39].

### Telomerase activity measurement

At 24 h after the start post-transfection, the cells were collected and lysed in CHAPS buffer. The telomeric repeat amplification protocol (TRAP) assay was performed using a modified version of the TRAPeze kit (EMD Millipore, Billerica, MA, USA), in which the primers and polymerase were added after the extension of the artificial telomere substrate. The PCR program was 30 cycles at 94 °C 30 s/59 °C 30 s/72 °C 45 s, and the products were run on a 15% polyacrylamide gel in 0.5× TBE (tris/borate/EDTA buffer), followed by staining with SYBR Gold Nucleic Acid Gel Stain (Invitrogen Life Technologies). The time course for telomerase activity was performed using the TRAPeze RT kit (EMD Millipore).

### Telomere length measurement using TRF

Genomic DNA was isolated using the TIANamp Genomic DNA kit (TIANGEN, Beijing, China) and run on an agarose gel to confirm its integrity, indicated as a tight crown-shaped band. Telomere restriction fragment analysis (TRF) was performed using the TeloTAGGG Telomere Length Assay (Roche, Basel, CH, Switzerland), and the resulting chemoluminescent Southern blot was imaged on a Tanon-5200 (Beijing Yuanpinghao Biotech, Beijing, China). The images were quantified to determine the mean telomere length using ImageJ.

### Senescence detection

β-galactosidase staining was performed using the Senescence β-galactosidase Staining Kit (Cell Signaling Technologies, Danvers, MA, USA).

### Karyotype analysis

The karyotypes of CD19-CAR T cells were analyzed using conventional Giemsa staining and G-banding with trypsin and Giemsa (GTG) at 14 days after TERT mRNA delivery. Briefly, cells were collected after 6 h of colcemid (100 ng ml^−1^) treatment and incubated sequentially in a hypertonic solution (0.075 M KCl) at 37 °C for 20 min, and then fixed in methanol/acetic acid (3:1) at room temperature for 20 min. The cell solution was dropwise added onto the slides. The Trypsin–Giemsa banding was carried out by Genetix GSL-120 automatic imaging system. And at least 10 metaphase cells were analyzed by the CytoVision Version 4.5.1 Build 5 software (Genetix, San Jose, CA, USA).

### Statistics

*T*-tests and correlation coefficient calculations were performed using the SPSS software for Windows Version 12.0 (SPSS, Chicago, IL, USA). The error bars represent the mean±s.d.
