# Enhancement of the *in vivo* persistence and antitumor efficacy of CD19 chimeric antigen receptor T cells through the delivery of modified TERT mRNA

**DOI:** 10.1038/celldisc.2016.32

**Published:** 2016-08-30

**Authors:** Yun Bai, Shifeng Kan, Shixin Zhou, Yuting Wang, Jun Xu, John P Cooke, Jinhua Wen, Hongkui Deng

**Affiliations:** 1 Department of Cell Biology and Stem Cell Research Center, School of Basic Medical Sciences, Center for Molecular and Translational Medicine, Peking University Health Science Center, Beijing, China; 2 The MOE Key Laboratory of Cell Proliferation and Differentiation, College of Life Sciences, Peking-Tsinghua Center for Life Sciences, Peking University, Beijing, China; 3 Shenzhen Stem Cell Engineering Laboratory, Key Laboratory of Chemical Genomics, Peking University Shenzhen Graduate School, Shenzhen, China; 4 Department of Cardiovascular Sciences, Center for Cardiovascular Regeneration, The Methodist Hospital Research Institute, Houston, TX, USA

**Correction to:** *Cell Discovery* (2015) **1**, 15040; doi:10.1038/celldisc.2015.40; published online 8 December 2015

In the initial published version of this article, there was an inadvertent omission from the Acknowledgements that the contribution of John P Cooke to this work was funded in part by a Core Facility Support Award from the Cancer Prevention and Research Institute of Texas (RP150611). This omission does not affect the description of the results or the conclusions of this paper.

