# Supplementary material for: Enhancement of the in vivo persistence and antitumor efficacy of CD19 chimeric antigen receptor T cells through the delivery of modified TERT mRNA
Source: Cell Discov. 2015 Dec 8;1:15040–. doi: 10.1038/celldisc.2015.40 (PMC4860832; doi:10.1038/celldisc.2015.40)
Supplement: Supplementary Information [file celldisc201540-s1.pdf]

Supplementary Materials and Methods

Western blot

The protein was harvested after washing the cells once with PBS, followed by lysing in RIPA buffer. The protein was subjected to electrophoresis on NuPAGE Novex Tris-Acetate Gels, followed by transfer to a PVDF membrane for 2 hours at 35 V, subsequently hybridized to anti-alpha tubulin at 1:1000 (Sigma, MO, USA) and anti-CD3-zeta antibody at 1:1000 or at 1:500 (Abcam, Cambridge, UK) and incubated overnight at 4°C. Secondary detection was performed using infrared (680 nm and 800 nm) antibodies (LI-COR) and the Odyssey imager (LI-COR). The total intensity of each band was quantified using the ImageJ program.

Hematoxylin-eosin stain

Different tissues of CAR-T-cell treated Raji NPG/Vst mice were inflated with 4% paraformaldehyde and fixed in the same fixative for 24 hours. Samples were processed and paraffin embedded. Serial sections were cut at 5 μm thickness and stained with hematoxylin and eosin (H&E). After examined by light microscopy, the tissues of model mice were infiltrated with a large number of lymphoma cells.

Supplementary Figure Legends

Figure S1. Generation of third-generation costimulatory CD19 CAR-modified T-cells and their in vitro immunobiology.

(A) Schematics of the pRRL-EF1A-19CAR3. This vector was modified with the EF1α promoter, and the anti-CD19 CAR comprises the FMC63-ScFv, the transmembrane and the intracellular domain of human CD28, activation domain of 41-BB and cytosolic signaling domains of CD3ζ. **(B)** The transfection efficiency of CD19 CAR lentiviral vector was demonstrated using flow cytometry at 72 hours after infection. T cells were stained with biotin-labeled polyclonal goat anti-mouse-F (ab')2 antibodies to detect the anti-CD19 CAR, and another sample of T cells were stained with biotin-labeled normal polyclonal goat IgG antibodies as an isotype control. **(C)** RT-PCR and Western blot illustrated the expression of CD19CAR in modified T cells at 72 hours after transfection. Western blot results from whole T-cell lysates probed with an anti-CD3-zata mAb. The unmodified T-cell line and CD19 CAR-modified T-cell clones displayed a 21-kDa band consistent with the wild-type CD3-ζ chain. In addition, CD19CAR-T cells showed a second band of approximately 70 kDa, consistent with the introduced chimeric CD3-ζ chain. **(D)** Surface molecule marker of the CD19CAR T cells after 2 weeks in vitro culture. **(E)** The cytokines secreted from CD19CAR-T cells after co-culture with $CD19^+$ tumor cells were detected using ELISA. The CD19 CAR T cells were co-cultured with $CD19^+$Raji tumor cells or $CD19^-$ K562 tumor cells at E:T ratios of 1:1, 10:1, and 25:1 in fresh medium without rhIL-2 for 4 hours, and subsequently the supernatants of these cultures were collected, centrifuged and detected by ELISA. The data are presented as the mean ± SD of results from 3 independent experiments. *$P < 0.05$, ***$P < 0.001$. **(F)** CD19 CAR-T cells show the specific tumor killing ability of $CD19^+$ tumor cells. Raji cells were $CD19^+$ human Burkitt's lymphoma cells, and K562 cells were $CD19^-$ human lymphoma cells. The target cells were incubated with CD19

CAR-specific $CD8^+$ T cells at E:T ratios of 1:1, 10:1, and 25:1, and after 4 hours of co-culture, the cytotoxicity of CD19 CAR-T cells was detected using a flow cytometric CTL assay. The data are presented as the mean ± SD of results from 3 independent experiments. *P < 0.05, ***P < 0.001.

Figure S2. Histologic analysis of tissue sections of sacrificed mice.

(A) Normal kidney tissue section of healthy NPG/Vst mice as control. **(B)** The kidney tissue section of CAR-T-cell treated Raji NPG/Vst mice model, all the tissues were conserved when the mice were dying. **(C)** Normal lung tissue section of healthy NPG/Vst mice. **(D)** The lung tissue section of CAR-T-cell treated Raji NPG/Vst mice. **(E)** Normal liver tissue section of healthy NPG/Vst mice. **(F)** The liver tissue section of CAR-T-cell treated Raji NPG/Vst mice. Scale bar was 100 µm.

Figure S3. Persistence of exogenous TERT mRNA and the telomere length in long-term culture.

(A) Persistence of exogenous hTERT mRNA were measured using RT-PCR after once independent electroporation at continuous interval time points. **(B**) The telomere length of TERT-CAR-T cells was initially measured at 48 h (2 days) after the third transfection, then the telomere length of TERT-CAR-T cells was measured every 10 days.

Figure S4. Cytokine release ability and cytolytic activity of TERT-CAR-T cells when co-cultured with $CD19^-$ K562 cells.

(A-D) Cytokine release of TERT-CAR-T cells was detected by ELISA after co-culture with

$CD19^-$ K562 cells at different E/T ratio (1:1, 10:1, 25:1), and four types of cytokines (IL-2, IL-10, IFN-γ, and TNF-α) were detected. The data are presented as the mean ± s.d. of results from 3 independent experiments. **(E)** CD19-specific CTL of TERT-CAR-T cells was detected by flow cytometry. **(F)** Cytolytic activity was determined after 4 hours of co-culture. The data are presented as the mean ± s.d. of results from 3 independent experiments.
