# Supplementary figures and images for: Enhancement of the in vivo persistence and antitumor efficacy of CD19 chimeric antigen receptor T cells through the delivery of modified TERT mRNA
Source: Cell Discov. 2015 Dec 8;1:15040–. doi: 10.1038/celldisc.2015.40 (PMC4860832; doi:10.1038/celldisc.2015.40)

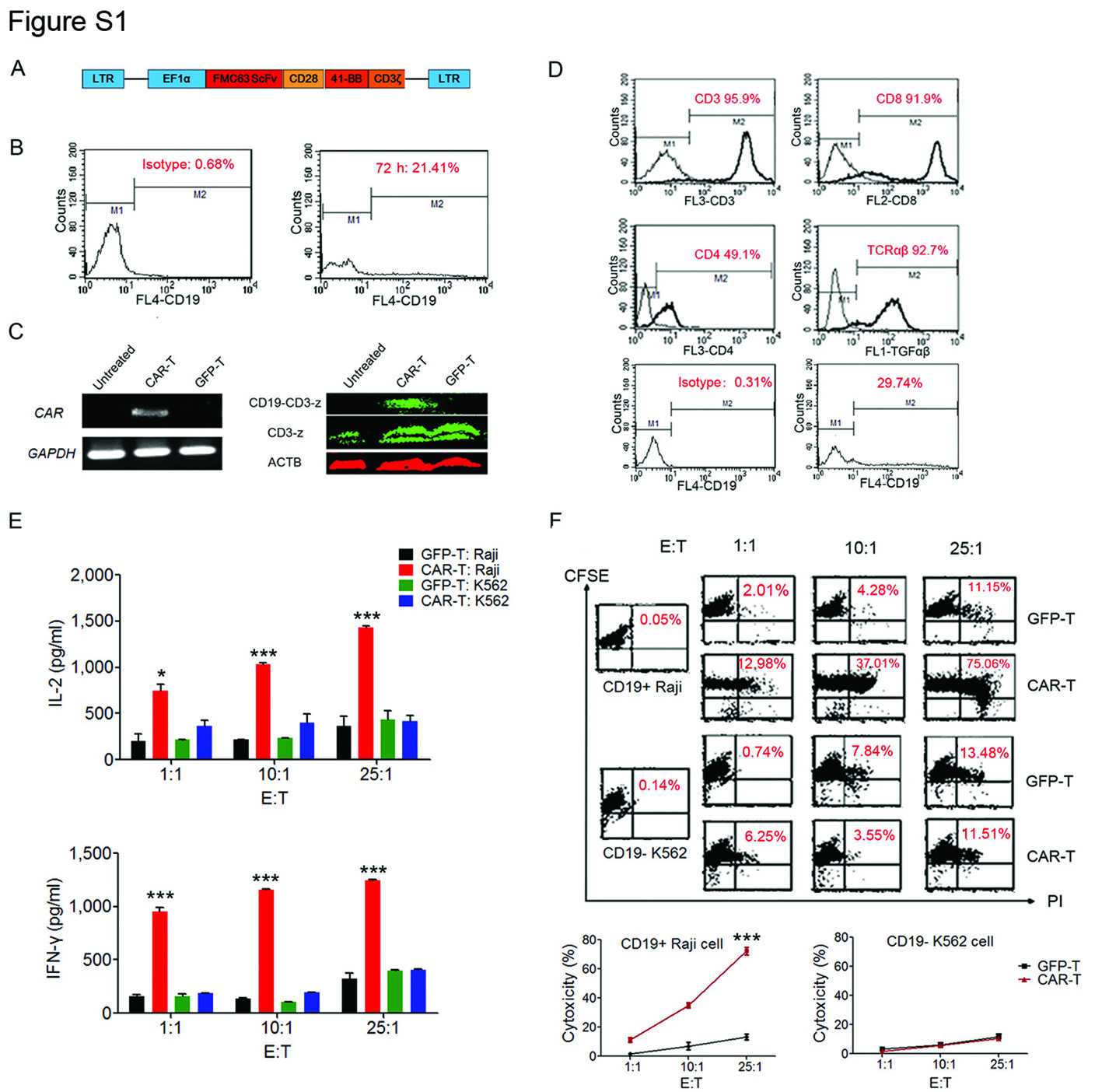

Supplement: Supplementary Figure S1 [file celldisc201540-s2.jpg]

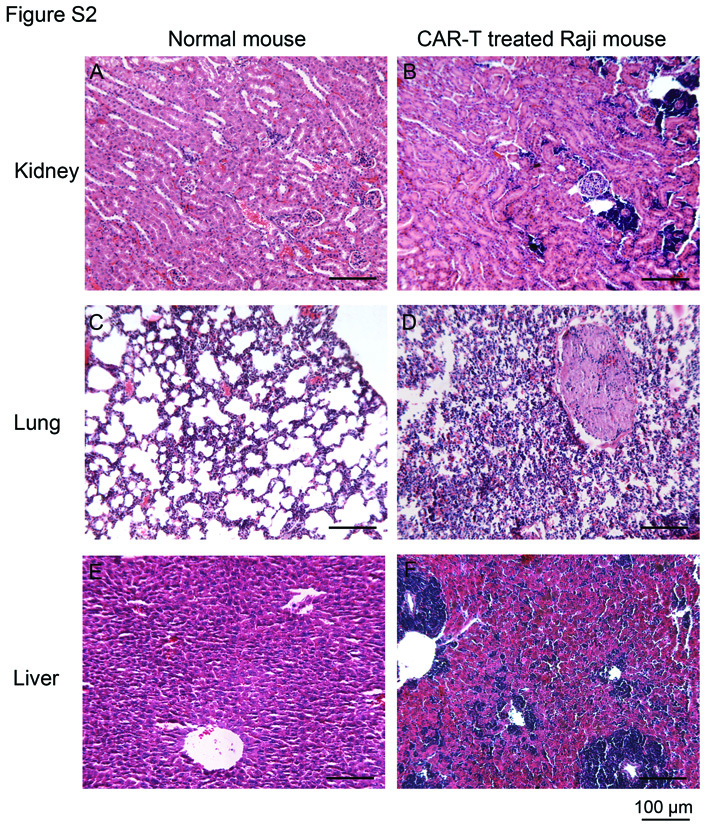

Supplement: Supplementary Figure S2 [file celldisc201540-s3.jpg]

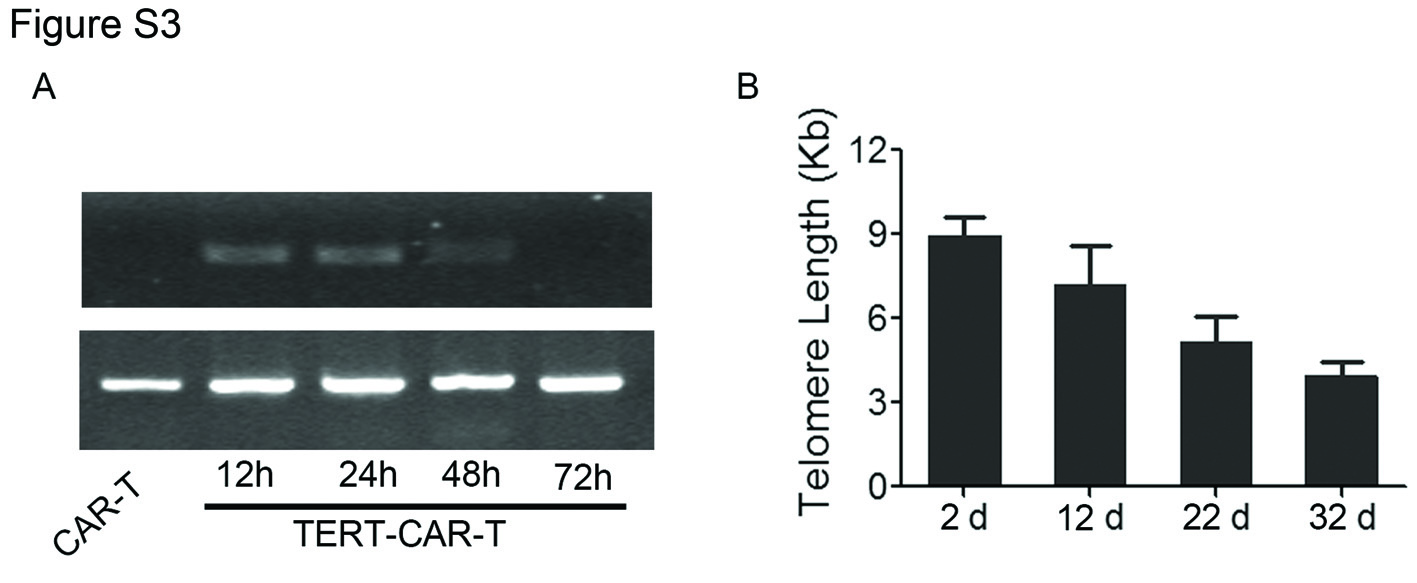

Supplement: Supplementary Figure S3 [file celldisc201540-s4.jpg]

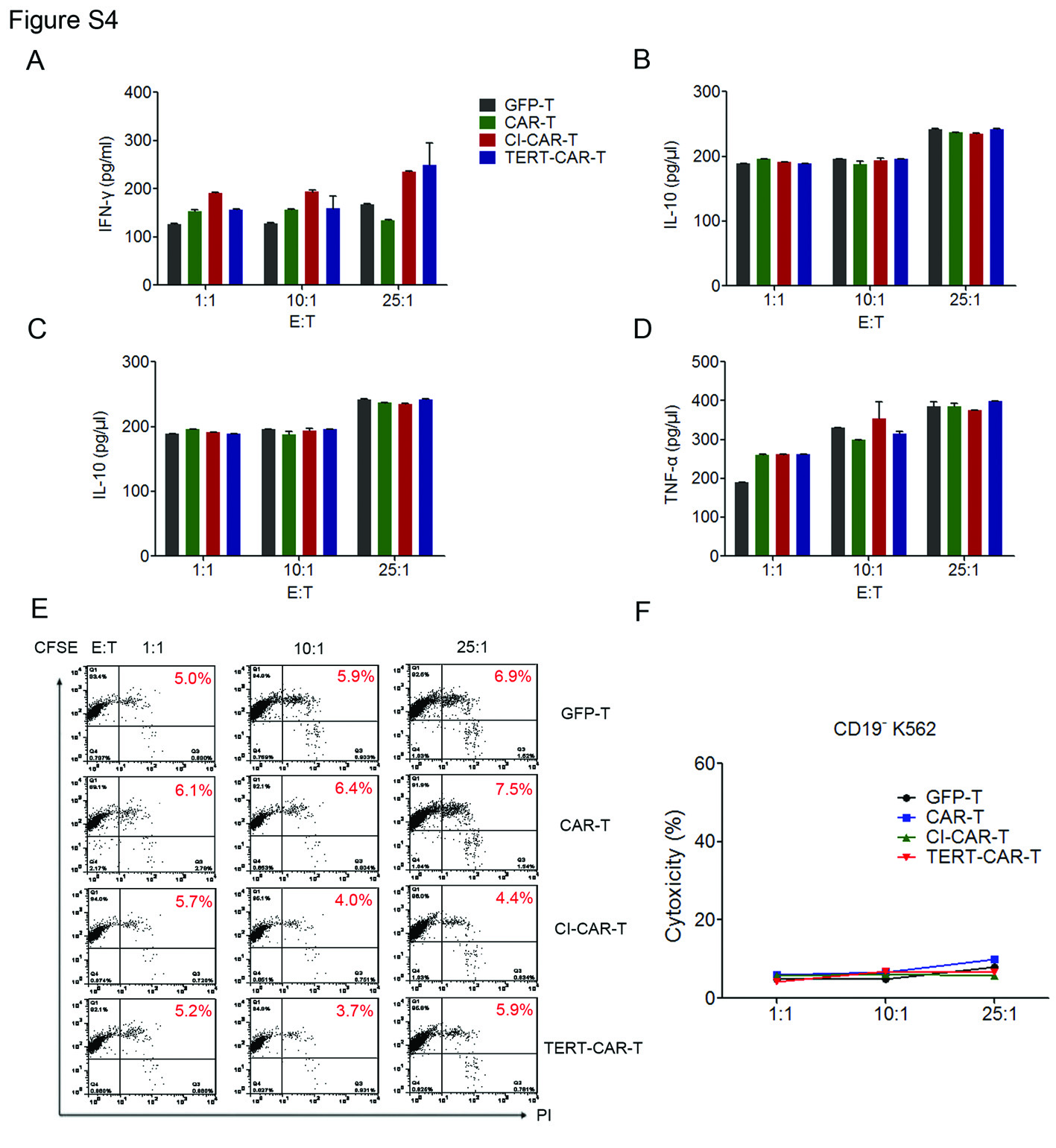

Supplement: Supplementary Figure S4 [file celldisc201540-s5.jpg]
